# Methodological panorama of clinical trials for diabetic foot ulcers: a scope review of design, implementation and reporting

**DOI:** 10.3389/fendo.2025.1710850

**Published:** 2025-11-24

**Authors:** Shuo Zhang, Yanying Wang, Ling Wang, Xuechun Fan, Jinyue Zhao, Yanyan Wang, Jing Yu, Yongjiang Yu, Yingjing Shi, Guanchi Yan, Jia Mi

**Affiliations:** 1 College of Traditional Chinese Medicine, Changchun University of Chinese Medicine, Changchun, China; 2 The Affiliated Hospital to Changchun University of Chinese Medicine, Changchun University of Chinese Medicine, Changchun, China; 3 Changchun Disabled Persons Rehabilitation Center, Changchun, China

**Keywords:** diabetic foot ulcer, clinical trial, study status, clinical design, scope review

## Abstract

**Background:**

Diabetic foot ulcer (DFU) reduce the quality of life for diabetes patients and creates a significant economic burden on global healthcare systems. Although there has been recent progress in DFU clinical trials, most existing scoping reviews have primarily focused on nursing or diagnostic methods. This review analyzed the characteristics of current DFU clinical trials and assessed research methods. This review focused on refining trial design and provide evidence-based methods for improving treatment outcomes and prognosis.

**Method:**

The study searched clinical trials registered in ClinicalTrials and Chinese Clinical Trial Registry (ChiCTR) (up to August 1, 2025). In addition, the study searched PubMed for clinical trials that were published from August 1, 2015, to August 1, 2025. Data was extracted and presented in tables.

**Result:**

The study revealed a rapid increase in the number of clinical trials focusing on DFU in recent years. Meanwhile, interventions have grown more varied, with skin transplantation, tissue replacement products, and dressings being widely used in clinical practice. Nonetheless, these studies face challenges, such as low methodological quality, lack of primary care studies, and low follow-up rates.

**Conclusion:**

The number of DFU clinical trials has increased, and the intervention strategies have become more varied. However, there is a large variation in the populations, design of the studies, and evaluation of their effectiveness.

## 1 Introduction

Diabetes Mellitus (DM) is a kind of metabolic disorder syndrome with chronic persistent hyperglycemia as the core pathological feature. In patients with diabetes (1), DFU is a major cause of disability and incapacity. The occurrence of diabetic foot ulcer globally is around 6.3% (2). About 25% of people with diabetes will experience foot ulcers at some point in their lives (3). About 42% of patients with diabetic foot have a recurrence within a year (4). Within five years, the recurrence rate can be as high as 65% (5, 6). Eventually, the vast majority of those with diabetic foot have difficulty curing the condition. Crucially, in the late stages of the disease, 20% of diabetic foot patients will face complications such as destruction of foot tissue and diminished neurovascular function. They have to undergo amputation, which results in losing their normal walking ability and significantly reduces their quality of life (7). Around 85% of non-traumatic lower limb amputations are due to diabetic foot (8).

The development of DFU is influenced by the interaction of high glucose environment, biofilm, inflammatory factors, neuropathy and vascular damage (9, 10). In recent years, the number of clinical trials registered for the treatment of diabetic foot has significantly risen. These interventions include skin transplantation and tissue replacement products, dressing products, stem cell therapy, and physical therapy. The healing rate for diabetes-related wounds remains at 30%, even with the best available treatment (11). It causes significant pain and financial burden for patients (12, 13).

Clinical trials play a crucial role in testing new therapies and refining existing programs. The quality of clinical trial design directly impacts the scientific validity and applicability of research outcomes (14). Clinical trial registration helps to reduce research waste and significantly boosts the quality of clinical research by enhancing transparency, preventing trial duplication, and optimizing resource distribution (15, 16).

This review used ClinicalTrials, ChiCTR, and PubMed as data sources. ClinicalTrials.gov is a clinical trial registration platform managed by the U.S. National Library of Medicine and the National Institutes of Health. This is the most extensive and reliable clinical research database globally, compiling data on both ongoing and completed studies worldwide (14, 17). Since its establishment in June 2007, ChiCTR has joined the World Health Organization’s International Clinical Trial Registry Platform (WHO ICTRP) as a registered institution (18). As the most extensively biomedical literature database, PubMed has greatly assisted researchers globally in conducting systematic reviews (19).

Although there are relevant reviews on the treatment, nursing and diagnosis of diabetic foot, there is a lack of a review of the clinical trial design and outcome of diabetic foot. Therefore, the heterogeneity of clinical trials in research design, intervention, diagnostic criteria and outcome restricts the integration and promotion of research results (20–22). The study aims to perform a scope review of the current clinical trial design status for diabetic foot, based on data from ClinicalTrials, ChiCTR, and PubMed. By optimizing the experimental design, the level of evidence in clinical research is improved (23).

## 2 Methods

### 2.1 Search strategy

In the search process of this study, the researchers (Shuo Zhang and Yanying Wang) conducted search for “diabetic foot”, “foot, diabetic”, “diabetic feet”, “feet, diabetic”, “foot ulcer, diabetic”, “diabetic foot ulcers” related research. The search period in ClinicalTrials and ChiCTR spanned from the inception of the database to August 1, 2025. PubMed was searched for clinical trials published between August 1, 2015, and August 1, 2025. At the same time, clinical trials and randomized controlled trials were selected on the pubmed article type filter to initially include clinical studies related to diabetic foot. Search strategy in **Supplementary Table 2**.

### 2.2 Inclusion and exclusion criteria

Inclusion criteria include: 1) Subjects: Patients diagnosed with DFU; 2) Interventions: To evaluate any interventions with adjuvant therapy as the main treatment for diabetic foot ulcers, including skin transplantation and tissue replacement products, dressing products, stem cell therapy, physical therapy, and Ethnic medicine treatment; 3) Study type: Interventional clinical trials, including randomized controlled trials, non-randomized controlled trials, single-arm trials; 4) Time range: For the literature published in PubMed, it is limited to the study published in the past 10 years (after August 1,2015).

Exclusion criteria are: 1) Studies not related to diabetic foot ulcers; 2) Diabetic foot treatments primarily based on surgery, such as amputation, and revascularization; 3) Patients with diabetic foot ulcers who underwent revascularization or amputation-related surgery; 4) Non-clinical trials (such as basic research, reviews); 5) Researches that cannot obtain full text or data cannot be extracted.

### 2.3 Data extract

We designed a standardized data extraction form using Microsoft Excel. Before the official extraction, two reviewers (Ling Wang and Xuechun Fan) used this form to pre-extract the characteristics of selected studies. Based on the results, the extraction form and extraction rules were calibrated and optimized to ensure consistent understanding among reviewers and feasibility of operation. The data extraction form covers the following core variables: number of registrations or publications, country, type of intervention, characteristics of study population, primary and secondary outcome measures, and study design. Items not classified into the above variables are uniformly marked as Others. Data extraction was completed independently by two reviewers (Ling Wang and Xuechun Fan). All extraction results were entered into the unified data extraction form. After the extraction was completed, the extraction results of the two reviewers were cross-checked. For data items with inconsistent extractions, the two reviewers first re-examined the original manuscripts and conducted discussions to reach a consensus. If the disagreement could not be resolved through discussion, a third senior researcher (Jia Mi) was invited to make a judgment, and this judgment was adopted as the final result.

### 2.4 Statistical analysis

Excel software was used for data analysis and processing. This study is a scope review of clinical research related to diabetic foot ulcers. Therefore, descriptive statistical methods are used to summarize and present the research characteristics extracted from the included literature in the form of frequency and percentage.

## 3 Results

### 3.1 Study selection and inclusion

**Figure 1:**
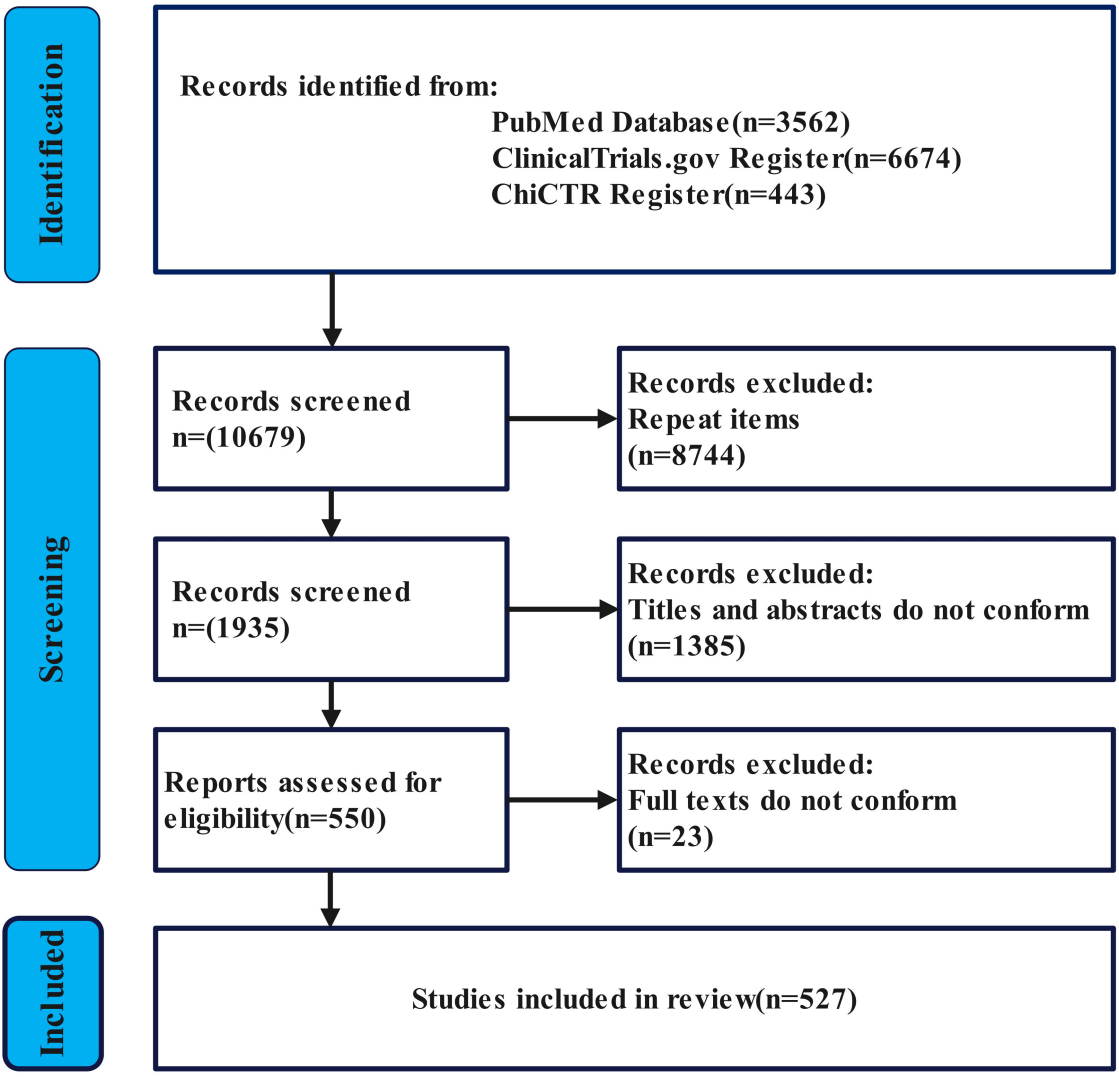
PRISMA search flowchart.

This study included a total of 527 clinical trials. The study retrieved a total of 10,679 records from the ClinicalTrials, ChiCTR, and PubMed databases. First, duplicate records were removed, excluding a total of 8,744 records. Subsequently, the remaining 1,935 records were screened, excluding 1,385 irrelevant studies. The remaining 550 records underwent full-text screening, among which 23 records did not meet the inclusion criteria. Finally, this study included a total of 527 clinical trials. Literature screening followed the PRISMA in **Figure 1** (24).

### 3.2 Number of registrations and publications, country

**Figure 2:**
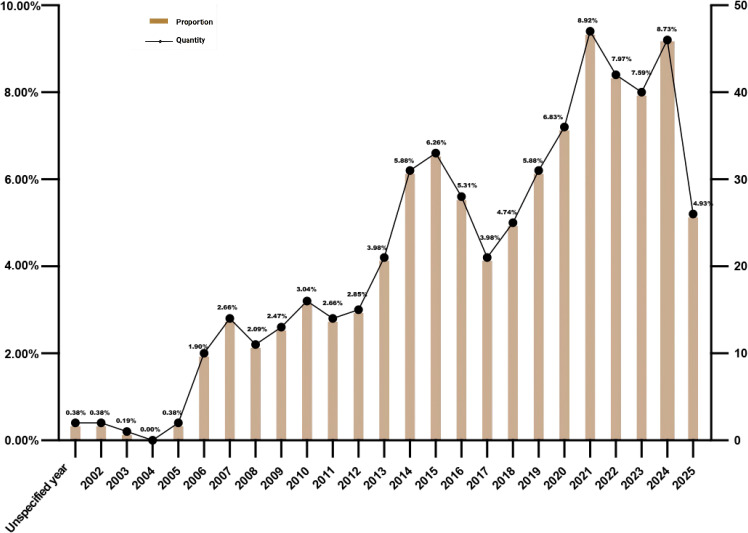
Annual number of registrations and publications: abscissa represents the year, left ordinate represents the proportion, and right ordinate represents the number.

**Figure 3:**
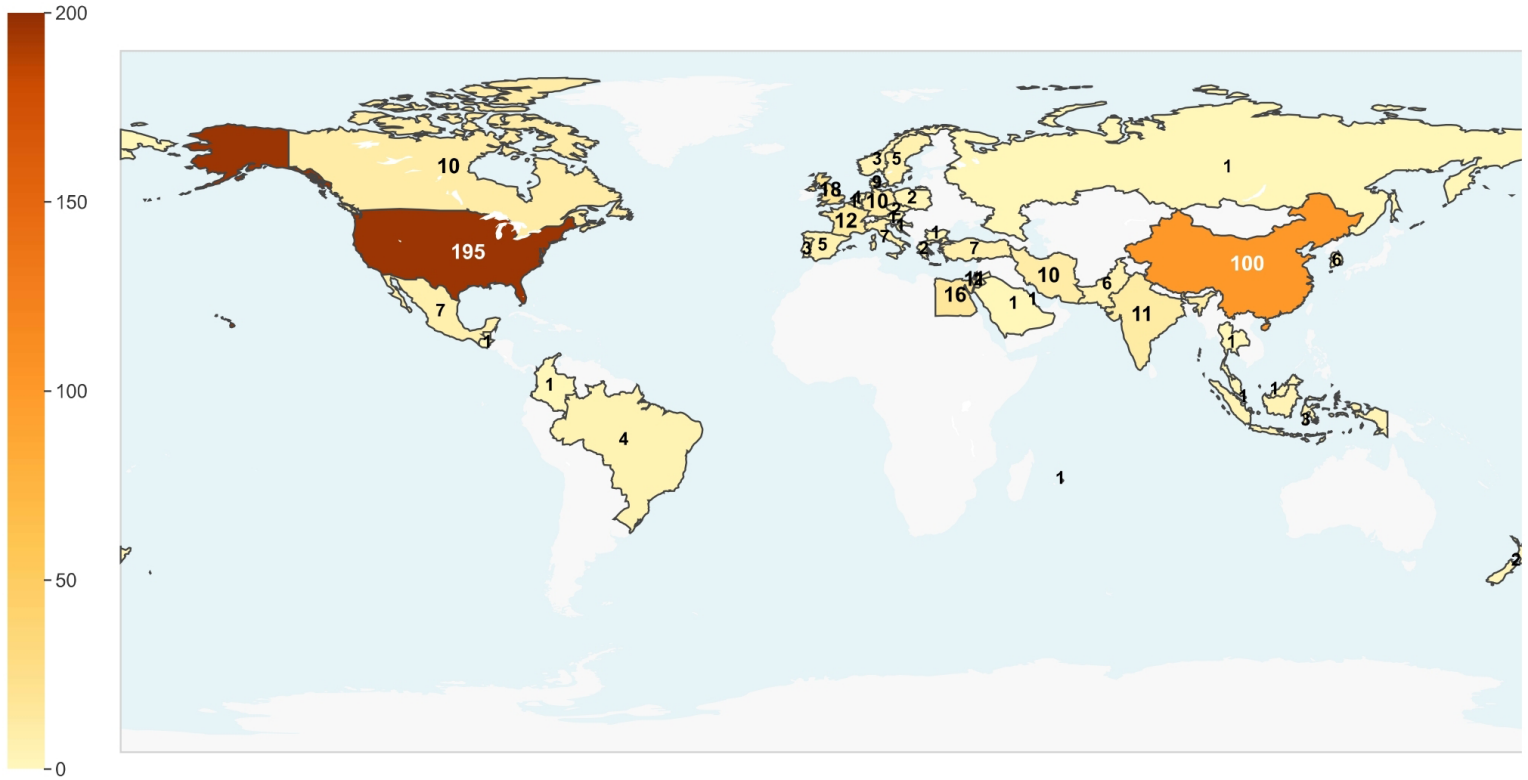
Published or registered country.

The overall evolution characteristics of the number of documents have changed from early low-level to significant growth in recent years. Before 2005, it was in the embryonic stage, with an average annual number of less than 5, and it was interrupted in 2004. From 2006 to 2013, it entered a stable growth period, and the number rose to double digits, from 10 to 21. Since 2014, it has entered a stage of rapid expansion, with a significant increase in the number, from 31 in 2014 to 36 in 2020, with an average annual high level, reflecting the continuous improvement of attention in this field. Since 2021, it has entered a high-level platform period, with the number of 40–47 in most years. Although it will fall back to 26 in 2025, the overall scale is still much higher than the historical level, indicating that clinical research in this field is highly active. The decline in 2025 data may be related to the lag in inclusion due to the inclusion deadline. In terms of the countries of registration or publication, the United States (195 studies) and China (100 studies) dominate the research landscape in this field, with the combined number of trials in the two countries accounting for 55.98% of the total. Although the United Kingdom, France, Germany and other countries steadily contribute evidence, the scale is limited (7–18 studies). While Middle Eastern countries such as Egypt, Iran, Israel, as well as countries like India and South Korea, exhibit remarkable research activity (6–16 studies). **Figure 2** showed the number of registrations and published clinical studies in different years. **Figure 3** showed the number of registrations and published clinical studies in different countries.

### 3.3 Characteristics of intervention

**Figure 4:**
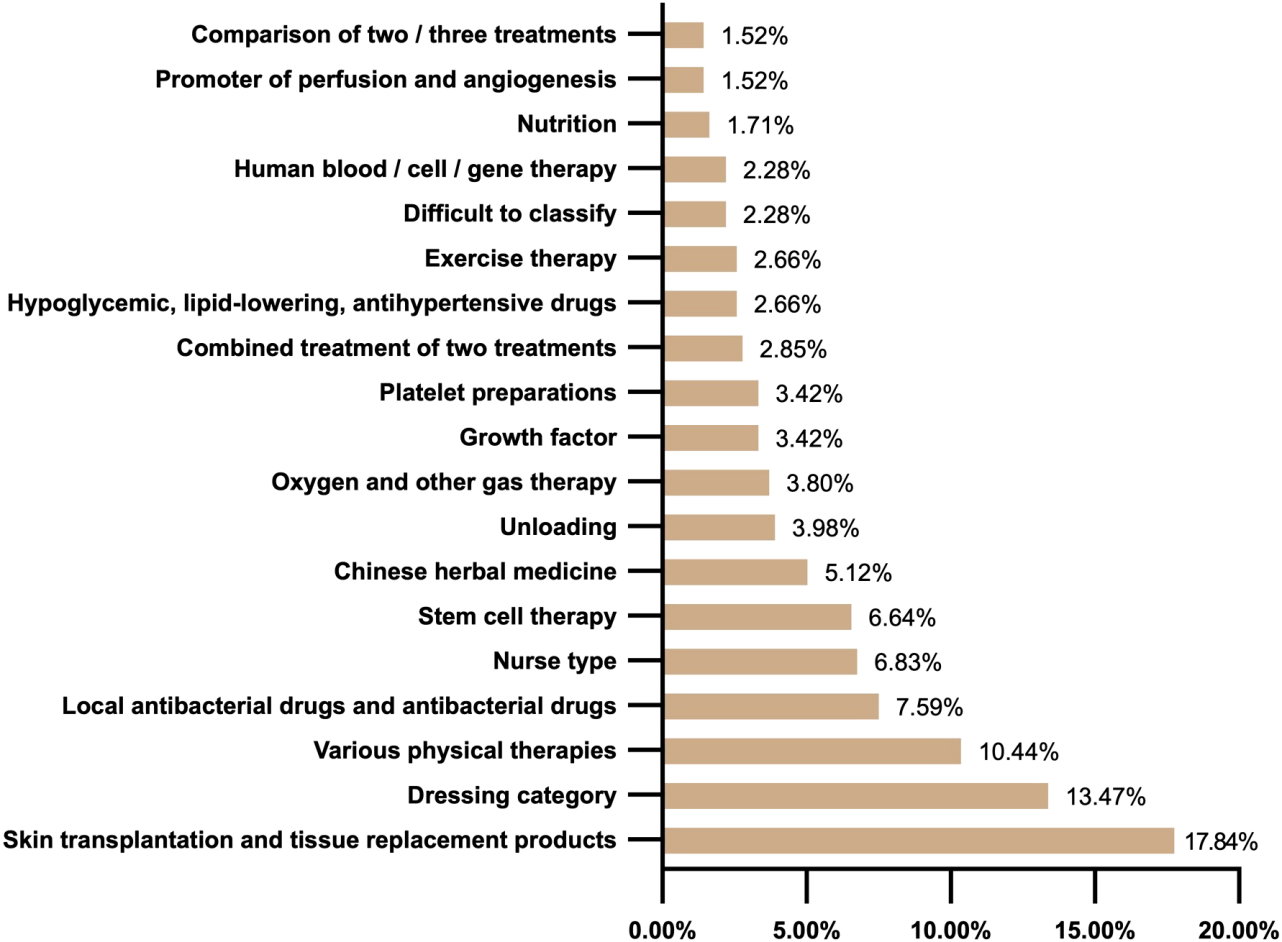
Classification of interventions.

The characteristics of intervention were shown in **Figure 4**. Skin transplantation and tissue replacement products had the highest frequency of intervention (94 studies, 17.84%), followed by dressing products (71 studies, 13.47%) and physical therapy (55 studies, 10.44%). In the field of biological therapy, stem cell therapy (35 studies, 6.64%), growth factors (18 studies, 3.42%) and platelet products (18 studies, 3.42%) showed certain research activity. In addition, Chinese herbal medicine treatment (27 studies, 5.12%) also occupies a certain proportion as a representative of complementary and alternative medicine. and interventions to improve local blood supply such as oxygen therapy (20 studies, 3.80%) and angiogenesis promoters (eight studies, 1.52%) are relatively low. In addition, some interventions such as human blood, cell, or gene therapy (12 studies, 2.28%) accounted for a relatively small proportion. However, in stark contrast to the activity of these interventions, the core basic treatment of diabetic foot ulcer management, such as decompression therapy (unloading device) related research is seriously insufficient, with only 21 studies (3.98%). There are few studies on nursing (36 studies, 6.83%) and exercise therapy (14 studies, 2.66%).only nine nutritional interventions, The frequency of attention to hypoglycemic, lipid-lowering and antihypertensive drugs (14 studies, 2.66%) was low.

### 3.4 Basic characteristics of population and ulcers

#### 3.4.1 Characteristics of population

In terms of the classification of diabetes in the population, there are significant problems of vague definition and insufficient stratification in current clinical studies. As many as 239 studies (45.35%) only included patients with type 1 or type 2 diabetes in a general way, while 233 studies (44.21%) did not even specify a specific type, and only “diabetic patients” were selected as the inclusion criteria. Notably, only 55 studies (10.44%) explicitly limited the population to the vast majority of patients with type 2 diabetes. This widespread unclear patient stratification is an important methodological limitation. Type 1 and type 2 diabetes may differ significantly in their pathophysiological mechanisms, disease progression, and systemic complications. Treating different populations together without differentiation will severely reduce the accuracy and interpretability of research results.

In ulcer grading, the Wagner grading system was dominant, with a total of 214 studies (40.61%) using this method. University of Texas classification was less used in 70 studies (13.28%). Another 23 studies (4.36%) used other grading methods or only grading but not clear grading methods. The other 220 studies failed to mention the grading method.

A total of 162 studies (30.74%) reported on the associated infection status of ulcers. In the remaining 365 studies, there was no mention of ulcer infection requirements.

In the assessment of vascular and neurological status, 110 studies (20.87%) clearly described the categories of ischemic, non-ischemic or neurological foot; another 274 studies (51.99%) clearly defined the blood perfusion of lower limbs through relevant examinations.

In terms of general condition assessment, blood glucose control was the most concerned indicator, and 198 studies (37.57%) put forward clear requirements for this; only 31 studies (5.88%) involved other laboratory examination indicators, and 15 studies (2.85%) put forward specific requirements for physical examination.

In the standard basic treatment measures, there were less descriptions of foot unloading requirements, a total of 120 studies (22.77%). Nursing-related descriptions were found in 57 studies (10.82%), while exercise behavior requirements were the least studied, only 19 studies (3.61%). **Table 1** shows the characteristics of population.

**Table 1:** Characteristics of intervention population.

Intervened group	Included literature	Record percentage (%)
Diabetes	233	44.21
Type 1 or type 2 diabetes	239	45.35
Type 2 diabetes	55	10.44
Wagner	214	40.61
Texas	70	13.28
Other classification methods	23	4.36
Ulcer infection/non-infection/both	162	30.74
Ischemic/non-ischemic/neurological foot	110	20.87
Lower limb blood perfusion examination	274	51.99
Indexes related to plasma glucose	198	37.57
Laboratory index	31	5.88
Physical examination	15	2.85
Unloading requirements	120	22.77
Nursing related requirements	57	10.82
Exercise behavior requirements	19	3.61
NA	32	6.07

#### 3.4.2 Basic characteristics of ulcer

**Figure 5:**
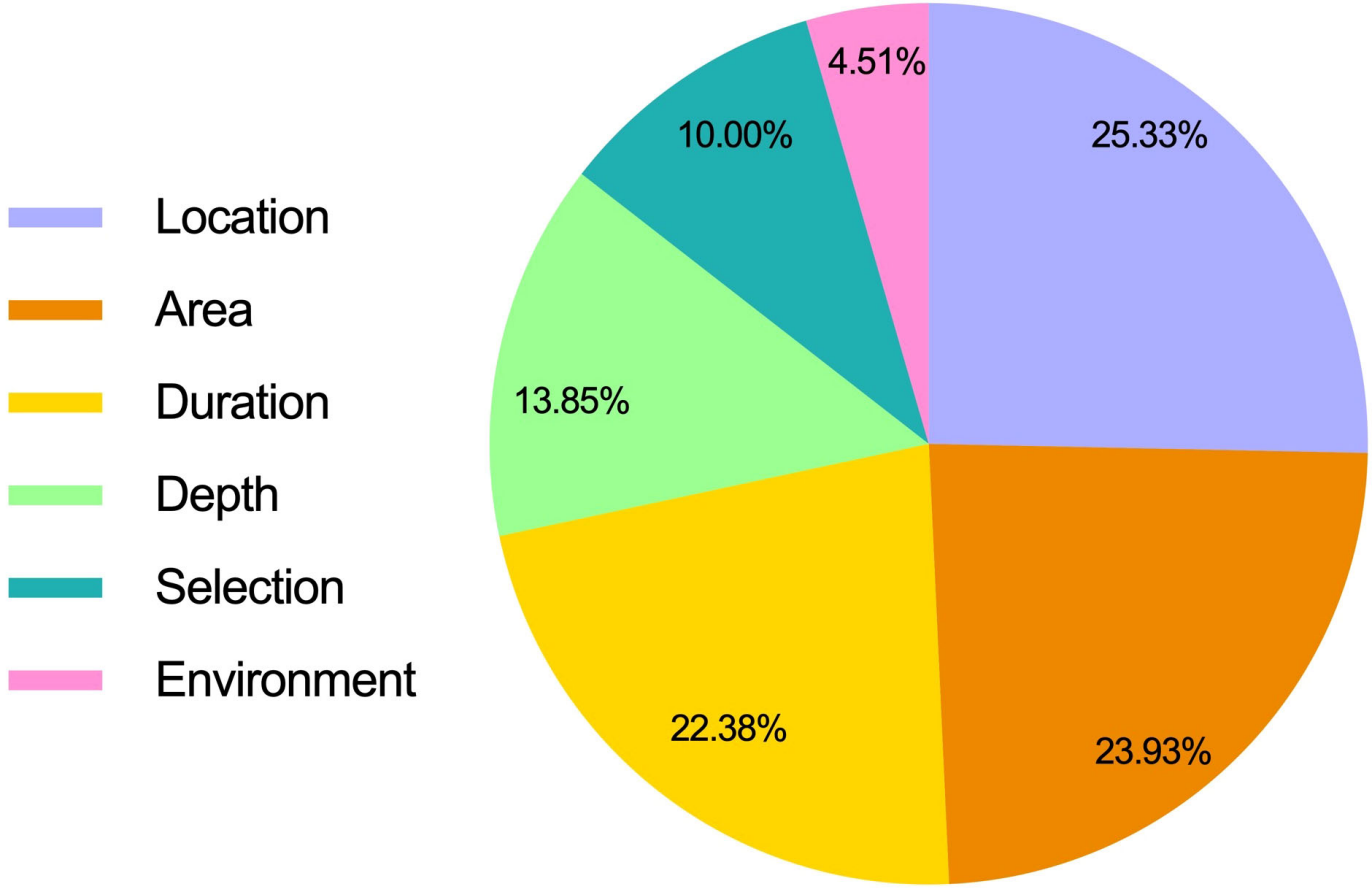
Characteristics of ulcer condition.

Among the 527 studies included, ulcer location (309 studies, 25.33%) and ulcer area (292 studies, 23.93%) were the most frequently reported indicators, and ulcer duration (273 studies, 22.38%) also received relatively more attention. However, ulcer depth, a key feature closely related to prognosis, was significantly underreported (169 studies, 13.85%). It is more noteworthy that there are few studies that clearly describe the selection criteria of target ulcers (122 studies, 10.00%), while studies that define the surface environment of ulcers (such as exudate, carrion, granulation tissue status, etc.) are even rarer (55 studies, 4.51%). The proportion of ulcer characteristics in the studies was shown in **Figure 5**.

### 3.5 Basic characteristics of studies

Within the included studies, the sample size of the patients was mainly concentrated in ≤ 50 (245 studies, 46.49%) and 51 to 100 (129 studies, 24.48%). There were 90 studies (17.08%) with a sample size between 101 and 200 people, 35 studies (6.64%) between 201 and 300 people, and only 25 large-scale clinical trials (4.74%) with a sample size of more than 300 people. Three other studies (0.57%) did not specify the sample size requirement.

In age distribution, most studies restricted patients to those aged 18 years and older (408 studies, 77.42%). There were significant differences in age standards in other studies, including 19 years old and above (five studies, 0.95%), 20 years old and above (40 studies, 7.59%), 30 years old and above (21 studies, 3.98%) and 40 years old and above (30 studies, 5.69%). The age criteria of 11 other studies were difficult to classify, and 12 studies (2.28%) did not specify the age requirement.

Gender was reported relatively completely in the studies. The vast majority of studies (524 studies, 99.43%) did not limit the gender of the subjects. Two studies restricted participants to males, and one study did not report gender requirements.

In intervention time, most of the intervention period were concentrated in 12 weeks and less (223 studies, 42.31%), followed by four weeks (including four weeks ± four days, 83 studies, 15.75%) and 24 weeks and less (67 studies, 12.71%). Another 69 studies did not report intervention time.

As many as 333 studies (63.19%) did not specify the follow-up period, and five studies (0.95%) did not set up follow-up. In the studies reporting follow-up, short-term follow-up within one month (49 studies, 9.30%) and medium-term follow-up within three months (65 studies, 12.33%) were the main ones, and only 11 studies (2.09%) carried out long-term follow-up for 12 months or more. The characteristics of clinical trial were shown in **Table 2**.

**Table 2:** Intervention characteristics of diabetic foot ulcer research.

Characteristics	Category	Included literature	Record percentage (%)
Sample size	0-50	245	46.49
	51-100	129	24.48
	101-200	90	17.08
	201-300	35	6.64
	Over 300	25	4.74
	Not specified	3	0.57
Age	18 years and older	408	77.42
	19 years and older	5	0.95
	20 years and older	40	7.59
	30 years and older	21	3.98
	40 years and older	30	5.69
	NA	11	2.09
	Not specified	12	2.28
Sexuality	All	524	99.43
	Male	2	0.38
	Not specified	1	0.19
Experimental intervention time	24 hours and within 24 hours	2	0.38
	1 week and 1 week ± 2 days.	10	1.90
	4 weeks and 4 weeks ± 4 days.	83	15.75
	8 weeks and within 8 weeks	52	9.87
	12 weeks and within 12 weeks	223	42.31
	24 weeks and within 24 weeks	67	12.71
	Over 24 weeks	21	3.98
	Not specified	69	13.09
Follow-up time	1 month and within 1 month	49	9.30
	3 months and less than 3 months	65	12.33
	6 months and within 6 months.	36	6.83
	12 months and within 12 months	28	5.31
	Over 12 months	11	2.09
	Not specified	333	63.19
	There was no follow-up	5	0.95

### 3.6 Basic characteristics of outcomes

In primary outcomes, the proportion of patient healing (178 studies, 33.78%) and the change of ulcer area (173 studies, 32.83%) were dominant. In contrast, ulcer healing time, the outcome with important clinical significance, was less concerned (55studies, 10.44%), while amputation events as key hard endpoints accounted for only 1.71%. In addition, the considerations of health economics and safety dimensions such as treatment cost (1.90%) and drug resistance (4.74%) are also limited.

In secondary outcomes, the system presents diversified characteristics. Changes in ulcer area (150 studies, 28.46%), healing time (137 studies, 26.00%) and healing ratio (117 studies, 22.20%) were widely used. The proportion of related adverse events (135 studies, 25.62%) and amputation (34 studies, 6.45%) was low. However, the cost and follow-up indicators were not given sufficient attention in the outcomes. The safety evaluation system was too single, relying heavily on generalized adverse event reports (220 studies, 41.75%). There was a lack of independent evaluation of safety outcomes including amputation (45 studies, 8.54%), safety, and drug resistance (45 studies, 8.54%). In addition, 120 studies (22.77%) did not set secondary outcomes. The outcomes of the clinical studies were shown in **Table 3**.

**Table 3:** Measurement of the results of diabetic foot ulcer research.

Characteristics	Category	Included literature	Record percentage (%)
Main evaluation index	Ulcer area changes	173	32.83
	Ulcer healing time	55	10.44
	Proportion of patients healed	178	33.78
	Related adverse events	58	11.01
	Questionnaire survey	48	9.11
	Laboratory examination	70	13.28
	Physical examination	20	3.80
	Safety and drug resistance	25	4.74
	Cost	10	1.90
	Follow-up	13	2.47
	Amputation	9	1.71
	Difficult to classify	12	2.28
	NA	4	0.76
Secondary evaluation index	Ulcer area changes	150	28.46
	Changes in ulcer volume	70	13.28
	Ulcer healing time	137	26.00
	Proportion of patients healed	117	22.20
	Related adverse events	135	25.62
	Wuestionnaire survey	135	25.62
	Laboratory examination	123	23.34
	Physical examination	23	4.36
	Safety and drug resistance	18	3.42
	Cost	26	4.93
	Follow-up	39	7.40
	Amputation	34	6.45
	Difficult to classify	20	3.80
	NA	120	22.77
Safety index	Adverse events	220	41.75
	Safety and drug resistance	45	8.54
	Amputation	45	8.54

### 3.7 Characteristics of clinical trials design

The current clinical research on diabetic foot ulcers presents a randomized controlled trial as main design method. In the allocation method, random allocation is dominant (431 studies, 81.78%). Non-random allocation accounted for only 3.04%. The intervention mode was mainly parallel distribution (400 studies, 75.90%), followed by single-component distribution (71 studies, 13.47%).

However, there are obvious deficiencies in the implementation of the blind method. As many as 45.73% of studies adopted an open-label design, while only 14.23% of studies adopted a double-blind design. In these trials, single-blind (77 studies, 14.61%), triple-blind (37 studies, 7.02%), and quadruple-blind (48 studies, 9.11%) designs were used. However, more than half of the studies did not adopt adequate blinding, which may have introduced significant differences.

In research scale, single-center studies accounted for 53.51%, and multi-center studies accounted for 41.37%. Among them, small and medium-sized studies with 2 to 15 centers were the majority (148 studies, 28.08%), and large clinical trials with 31 or more centers accounted for only 2.85%.

In number of test groups, the double-arm design was absolutely dominant (345 studies, 65.46%), followed by the single-arm design (72 studies, 13.66%), and the complex design (three-arm and above) was relatively less. In addition, the proportion of studies using placebo control was low (100 studies, 18.98%). The study design of clinical trials was shown in **Table 4**.

**Table 4:** Research design of diabetic foot ulcer.

Characteristics	Category	Included literature	Record percentage (%)
Allocation	Randomized	431	81.78
	Non-randomised	16	3.04
	NA	64	12.14
	Queue	5	0.95
	Case-Control	4	0.76
	Case-Only	4	0.76
	Unknown	3	0.57
Intervention model	Single Component	71	13.47
	Parallel	400	75.90
	Cross-over	7	1.33
	Sequential Assignment	6	1.14
	Factorial Assignment	3	0.57
	Unknown	40	7.59
Masking/Blinding	Single blind	77	14.61
	Double blind	75	14.23
	Triple blind	37	7.02
	Quadruple blind	48	9.11
	Open Label	241	45.73
	Unknown	48	9.11
	NA	1	0.19
Participating center	Single center	282	53.51
	The number of multi-centers is not clear	16	3.04
	Multicenter 2-15	148	28.08
	Multicenter16-30	39	7.40
	Multicenter 31 or more than 31	15	2.85
	Unknown	27	5.12
Placebo comparator	Yes	100	18.98
	No	427	81.02
Set the number of test groups	Single-arm test	72	13.66
	Two arms test	345	65.46
	Three-arm test	52	9.87
	Four-arm test	24	4.55
	Five arms test	8	1.52
	Six-arm test	5	0.95
	Unknown	21	3.98

## 4 Discussions

This study reviews the current status of clinical trials to provide a reference for researchers to comprehend the trial design of diabetic foot ulcers. Previous studies have focused on care and diagnosis (25, 26). Yet, there is a lack of a scope review of clinical trials aimed at diabetic foot ulcers. This is the first comprehensive evaluation of the characteristics of clinical trials related to diabetic foot ulcers. This review indicates that over the past 20 years, clinical research on diabetic foot ulcers has transitioned from sporadic studies to consistent development. Additionally, as a major diabetes complication, there have been fundamental changes in clinical care and research funding (27, 28).

There is a trend towards diverse development in interventions for diabetic foot ulcers in clinical studies. Among these interventions, the number of clinical trials related to skin transplantation and tissue replacement products ranks first. This reveals that these bioengineering treatments are being increasingly appreciated and are gradually applied in medical practice. This trend is consistent with the recent review of the field of diabetic foot treatment (7). Current research indicates that these tissue engineering products are a preferred option for enhancing ulcer closure and healing in diabetic foot ulcer patients (29). At the same time, dressing treatments has the advantages of simple operation, diverse functions, and strong ability to regulate the wound microenvironment. The rapid development of these two treatment strategies reflects the dual focus of researchers in this field on wound structure repair and precise regulation of the microenvironment (9).It is worth noting that stem cell therapy, as a representative of regenerative medicine, has shown research activity (30), and together growth factors and platelet products, constitutes a research cluster for innovative therapies. According to guidelines, unloading therapy is consistently recommended as core measure in the treatment of diabetic foot ulcers (31). Nonetheless, clinical research on offloading devices is considerably lacking, with just 21 studies focusing on this. The phenomenon was further confirmed by the analysis of population characteristics. Only 22.77% of studies clearly described the requirement for unloading, which is seriously inconsistent with its key role in clinical practice and guidelines. This distribution pattern reveals a potential innovation bias in existing clinical research. This means an overemphasis on novel biomaterials and technologies while neglecting fundamental treatment strategies strongly recommended in guidelines as the cornerstone of treatment. This neglect of fundamental treatments may lead to bias in evidence-based medicine. Despite increasing data on interventions, there is a lack of robust evidence to guide the optimal implementation and optimization of fundamental treatments such as decompression and care. This imbalance complicates the development of comprehensive and practical treatment guidelines because it is difficult to accurately assess the synergistic effects between core and adjunctive innovative treatments. Future research needs to rebalance its focus and strengthen the scientific validation and optimization of fundamental treatment strategies.

Another key methodological problem revealed in this review is the heterogeneity and imprecision of population definition. Results showed that nearly 90% of trials failed to accurately distinguish specific types of diabetes. Given the fundamental differences in ulcer pathology and amputation rates between patients with type 1 and type 2 diabetes (32). Pooling these two heterogeneous populations presents two major drawbacks. First, it significantly reduces the reproducibility of studies, making it difficult for subsequent researchers to determine whether the patient groups truly match the original study. Second, and more importantly, it hinders our ability to accurately assess the differences in the efficacy of interventions for different diabetes subtypes, potentially masking important signals of effectiveness or ineffectiveness in specific populations. This research paradigm is one of the key reasons why current evidence on DFU is difficult to integrate and translate into precise clinical practice. Future research must adopt a clear diabetes classification and patient stratification based on pathophysiological characteristics as standard methods. In ulcer grading, Wagner classification remains the most prevalent, whereas the more detailed Texas classification has been applied in just 70 studies. As the earliest grading method, the Wagner classification has the advantage of being simple and easy to use. However, it primarily focuses on ulcer depth and has the limitation of ignoring ischemia and infection, two key factors that influence prognosis. In contrast, Texas classification considers the three dimensions of lesion depth, infection and ischemia (33). It provides a more comprehensive assessment framework and selection of individualized treatment strategies. This review recommends that researchers choose a grading system that is consistent with their study objectives during the design phase and provide instructions for its application in their reports. Furthermore, the combination of different grading systems can present a more comprehensive picture of ulcer characteristics.

In general condition assessment, current research shows obvious imbalance. There is strong focus on controlling blood glucose, yet other laboratory indicators and physical examinations are considerably neglected. Overall, current research on diabetic foot ulcers suffers from unclear disease classification, inconsistent grading systems, and insufficient attention to general condition. These problems greatly undermine the scientific credibility, feasibility, and comparability of studies. This is one reason why, despite a wealth of research, the prognosis assessment for diabetic foot ulcers remains constrained (11).

This review highlights the prevalence of small-scale and limited-follow-up studies in current clinical trials. 70.97% of trials included fewer than 100 participants, and only 4.74% had a sample size exceeding 300. Given amputation as a hard endpoint with a significant impact on patients’ quality of life, small-sample trials lack sufficient statistical power to detect even small but clinically significant risk reductions from treatment interventions. Most studies (42.31%) reported intervention durations of 12 weeks or less, suggesting that 12 weeks may be a critical time point for efficacy assessment. A more pressing issue is the excessively short follow-up period and the general lack of emphasis on establishing such follow-up assessments. DFU have a high recurrence rate, with literature reporting a one-year recurrence rate as high as 42% (4). However, only 2.09% of the studies included in this study had a follow-up period exceeding one year, which is insufficient to effectively assess the long-term value of treatment in preventing recurrence. This design flaw leads to an over-reliance on surrogate endpoints, such as ulcer size reduction or short-term healing rates, in the current evidence framework, severely neglecting outcomes equally crucial to patients and the healthcare system: sustained healing, reduced recurrence rates, and long-term cost-effectiveness. Compared to other methodological limitations, clinical studies of diabetic foot ulcers have demonstrated relatively high completeness and inclusion in gender reporting. In summary, current clinical research on diabetic foot ulcers is affected by various methodological deficiencies. In conclusion, current clinical trials of diabetic foot ulcers generally suffer from small sample sizes and severely inadequate follow-up periods. While this short, small-scale study model is common in exploratory research, it significantly limits the statistical power and clinical extrapolation value of its results. Due to insufficient statistical power, these studies may fail to detect the true differences in hard endpoints (such as amputation) caused by interventions. Furthermore, the excessively short follow-up period fails to capture the high recurrence rate of diabetic foot ulcers, thus reducing our ability to observe the long-term efficacy and recurrence prevention capabilities of interventions. Therefore, future research should prioritize larger-scale, longer-term study designs. Advocating a shift to a large-scale, long-term research paradigm aims not only at improving methodology but also at addressing the lack of high-quality evidence, ultimately shifting the cornerstone of clinical decision-making from statistically significant changes to tangible improvements in patients’ quality of life.

Currently, the selection of outcome measures in clinical trials for diabetic foot ulcers suffers from serious structural flaws, primarily manifested in a lack of standardization and insufficient clinical relevance. Compared to the core outcome measure set for diabetic foot ulcers, existing studies show a significant directional bias (12), Currently, there is an over-reliance on surrogate endpoints, such as changes in ulcer area (32.83%) and patient healing rate (33.78%), while crucial hard endpoints for both patients and the healthcare system, including amputation (accounting for only 1.71% as the primary endpoint) and ulcer healing time (10.44%), are systematically ignored. Treatment costs, reflecting the utilization of healthcare resources, account for only 1.90%. This result is inconsistent with the cost expenditures emphasized in the guidelines (34). This indicates that clinical trials in this field often neglect economic benefits. This disconnect between research focus and actual clinical needs results in numerous studies producing insufficient evidence to guide clinical decision-making and health policy development. The selection of secondary outcome measures and the safety assessment system further highlight the fragility of the evidence base. Although the proportion of amputation (6.45%) and cost (4.93%) as secondary outcome measures has increased slightly, they remain marginalized. More worryingly, safety assessments rely excessively on generalized adverse event reports (41.75%), lacking independent assessments of key safety endpoints such as amputation (8.54%) and drug resistance (8.54%). Furthermore, 22.77% of studies did not even include secondary outcome measures, reflecting a systematic neglect of comprehensive efficacy assessment. The imbalance in outcome measure selection severely limits the practical value of research. Clinicians are unable to assess the substantial impact of interventions on amputation risk based on existing evidence; health insurance institutions lack reliable long-term cost-effectiveness data; and patients’ core concerns, such as sustained wound healing and improved quality of life, are not adequately addressed. Therefore, we strongly recommend a fundamental paradigm shift in future research on diabetic foot ulcers, mandating the use of a core outcome measure set that includes amputation rate, ulcer recurrence rate, treatment cost, and patient-reported outcomes. This will help establish a high-quality evidence-based system that truly guides clinical practice and improves patient outcomes.

In these clinical studies, 81.78% employed randomization, demonstrating the emphasis on maintaining high evidence quality and methodological standards. Many outcomes for diabetic foot ulcers are based on subjective assessments. Without blinding, researchers might inadvertently bias the outcome towards their expectations, thereby introducing measurement bias (35, 36). However, there were significant weaknesses in blinding implementation, which may be stem from the challenges of applying it in some trials. In terms of research scale and center participation, single-center research still dominates (53.51%), while large-scale clinical trials involving 31 or more centers only account for 2.85%. This reflects significant opportunity for improvement in multicenter collaboration. Strengthening cross-center collaboration will help improve research efficiency, reduce resource waste and enhance results extrapolation. The restricted application of placebo in these studies (only 18.98%) reflects the unique ethical dilemmas and the scientific challenges of intervention design. Delaying effective treatment for diabetic foot ulcers may result in irreversible damage, including wound progression, increased risk of infection, and amputation. Therefore, the use of placebo control not only involves significant ethical controversy, but is also often unfeasible because it is difficult to design a credible placebo.

In general, the current clinical research still faces a variety of methodological challenges in design, including insufficient application of blind methods, dominance of single-center studies, and limited use of placebos. These problems are intertwined, which restricts the reliability and generalization of the research results. These limitations may introduce significant performance and detection biases, thereby systematically affecting the objectivity of efficacy evaluation and ultimately undermining the reliability of the entire evidence system. Future research should further improve the awareness of methodological quality, and describe methodological details comprehensively and transparently in program design and report.

As a scope-defined review, this study aims to describe the distribution and characteristics of existing evidence, rather than to assess the risk of bias or classify evidence for individual studies, the latter being the core task of systematic reviews. Similarly, this paper also has certain limitations. First, publication bias may exist, such as incomplete registration data for some trials, or the exclusion of unpublished literature or non-English-language studies. Second, despite the comprehensive search strategy, relevant trials may still be missed, leading to selection bias and publication bias.

## 5 Conclusion

This study is the first systematic review of the current state of clinical research on diabetic foot ulcers, revealing key methodological limitations in current trial designs. In the future, conducting systematic evaluations and meta-analyses of various interventions will be essential to offer strong evidence for optimizing treatment strategies.

## Data Availability

The original contributions presented in the study are included in the article/**Supplementary Material**. Further inquiries can be directed to the corresponding authors.

## Supplementary material

Supplementary Table 1 Included papers information, Supplementary Table 2 Search strategy.
