# Supplementary material for: Methodological panorama of clinical trials for diabetic foot ulcers: a scope review of design, implementation and reporting
Source: Front Endocrinol (Lausanne). 2025 Nov 24;16:1710850. doi: 10.3389/fendo.2025.1710850 (PMC12682631; doi:10.3389/fendo.2025.1710850)
Supplement: Supplementary file 1 [file Table2.docx]

**Supplementary Table 2 Search strategy**

| **The search strategy for PubMed** | |
| --- | --- |
| **Number** | **Search terms** |
| #1 | (Diabetic Foot OR Foot, Diabetic OR Diabetic Feet OR Feet, Diabetic OR Foot Ulcer, Diabetic OR Diabetic foot ulcer) |
| #2 | (clinical trials OR randomized controlled trials) |
| #3 | #1 AND #2 |

| **The search strategy for** **Clinicaltrials** |
| --- |
| (Diabetic Foot OR Foot, Diabetic OR Diabetic Feet OR Feet, Diabetic OR Foot Ulcer, Diabetic OR Diabetic foot ulcer) |
| **The search strategy for ChiCTR** |
| (Diabetic Foot OR Foot, Diabetic OR Diabetic Feet OR Feet, Diabetic OR Foot Ulcer, Diabetic OR Diabetic foot ulcer) |
